# Acute, chronic, and non-fracture-related compartment syndrome in children. A current concepts review

**DOI:** 10.1177/18632521261485657

**Published:** 2026-09-03

**Authors:** Francesco Candusso, Sara De Salvo, Camilla Cabrioli, Loris James Bari, Roberto Facchi, Emanuela Lanari, Marco Natale, Federico Canavese

**Affiliations:** 1 Dept. of Orthopedics, 18572 IRCCS Istituto Giannina Gaslini, Genova, Italy; 2 Department of Medical and Surgical Specialties, Radiological Sciences, and Public Health, University of Brescia, Brescia, Italy; 3 DISC - Department of Surgical Sciences and Integrated Diagnostics, University of Genova, Genova, Italy

**Keywords:** compartment syndrome, pediatric, children, fasciotomy, Volkmann’s contracture, intracompartimental pressure

## Abstract

**Background:**

Pediatric acute compartment syndrome (PACS) is a rare limb-threatening emergency. Diagnosis is complicated by pediatric developmental physiology and communication barriers. Although traumatic fractures are the primary cause, non-fracture etiologies present unique diagnostic challenges. This review summarizes the latest evidence on the changing epidemiology, pathophysiology and diagnostic approaches to acute, chronic, and non-fracture compartment syndromes in children.

**Methods:**

A comprehensive literature search was conducted across the PubMed/MEDLINE, Embase, and the Cochrane Library databases. The review included English-language, peer-reviewed articles published between January 1966 and May 2026, utilizing Medical Subject Headings and keywords such as “compartment syndrome,” “pediatric,” “children,” and “fasciotomy”.

**Results:**

The diagnostic approach to PACS has shifted from the unreliable “5 Ps” at late stages to the early “3 As”: increased anxiety, agitation, and analgesic requirements. Children naturally have higher baseline compartment pressures than adults, so pediatric-specific interpretations of pressure monitoring are required. While fracture-related PACS generally has an excellent prognosis if recognised and treated promptly, non-fracture acute compartment syndrome frequently suffers from critical diagnostic delays, leading to high rates of myonecrosis. The definitive standard of care remains an emergent decompressive fasciotomy. However, pediatric tissue demonstrates robust regenerative capacity, enabling conservative initial muscle debridement and achieving high success rates with delayed primary wound closure.

**Conclusions:**

Although pediatric patients have remarkable healing potential, even following delayed surgical interventions, early recognition is essential to preventing permanent disability. The ability to safeguard limbs depends entirely on clinician’s ability to prioritize the behavioral “3 As” over late-stage neurovascular changes.

## Introduction

The most common cause of compartment syndrome in children is trauma.^1,2^ Acute compartment syndrome (ACS) is an orthopedic emergency that threatens the limb. It requires a high level of clinical suspicion and immediate decompression. Because it is time-sensitive, failure to promptly recognize and treat it can lead to severe complications.^3,4^ A missed diagnosis can result in Volkmann’s ischemic contracture, a devastating, lifelong disability.^5,6^ However, the developmental physiology of growing individuals and the communication barriers of the pediatric population can complicate diagnosis.^7,8^ Additionally, since ACS is less common in children than in adults, clinicians who work primarily with children may not recognize its signs and symptoms.^7,9^ Moreover, studies indicate that the time between injury and development of peak compartment pressure in children may exceed 24 hours, which is significantly longer than in adults.^8,10^

These factors can lead to prolonged ischemic periods and adverse outcomes because the core pathophysiology involves critical elevation of intracompartmental pressure (ICP) within an inelastic fascial space. This leads to microvascular compromise via alteration of the arteriovenous gradient, resulting in progression of hypoxia and cellular damage. Ultimately, this can result in irreversible muscle necrosis and nerve death.^1,6,11,12^ Timely recognition and surgical decompression are critical to saving the limb and its function.

While fractures are the primary concern for clinicians, there is a growing recognition of non-fracture etiologies that present unique diagnostic challenges. Other causes of compartment syndrome include vascular injuries, surgical positioning, extravasation, infections, and insect, snake, or animal bites.^1,3^ Additionally, in developing nations, treatments by traditional bone setters represent an additional cause of pediatric compartment syndrome.^13,14^ ACS can affect children of any age, though the etiology correlates with age.^1,15^ In children under 10 years of age, the cause is usually a vascular injury or infection. In children over 14 years of age, trauma or surgical positioning is typically the cause.^1,16^

This review aims to synthesize the latest evidence and help clinicians navigate the evolving epidemiological landscape and diagnostic paradigms of acute, chronic, and non-fracture-related compartment syndrome in children.

## Methods

A comprehensive literature search was conducted across the PubMed/MEDLINE, Embase, and Cochrane Library databases. The search identified articles relevant to ACS in the pediatric population that were published between January 1966 and May 2026. The search utilized combinations of the following Medical Subject Headings (MeSH) and free-text keywords: “compartment syndrome”, “pediatric”, “children”, “fracture”, “intracompartmental pressure”, and “fasciotomy”. The search was restricted to English-language and peer-reviewed articles.

## Epidemiology and pathophysiology of growing limbs

The vast majority of pediatric acute compartment syndrome (PACS) cases are caused by high-energy trauma.^11,17^ Non-traumatic causes, on the other hand, include vascular injuries, surgical positioning, infections, envenomation, and restrictive treatments applied by traditional bone setters. These causes fall under the umbrella of Non-Fracture Acute Compartment Syndrome (NFACS).^1,14,18,19^

PACS can occur at any time during childhood and adolescence. However, the risk profiles and primary etiologies differ significantly between early and late adolescence, reflecting the anatomical and physiological changes that occur during musculoskeletal maturation.^1,20^ In children under 10 years of age, the cause is usually a vascular injury or infection. In children over 14 years of age, trauma or surgical positioning is typically the cause.^9,16,21^ The risk profiles for PACS are summarized in Table 1, which categorizes them by age group (Table 1).

**Table 1.** Risk profiles for pediatric acute compartment syndrome, categorized by age group.

Feature	Early childhood (<10 years)	Adolescence (≥14 years)
Primary Etiologies	Vascular insults, IV infiltration, infection	High-energy trauma, overexertion, sports
Risk Factors	Iatrogenic injury, systemic illness	High BMI, comminuted/segmental fractures
Anatomical Drivers	Developing vascular systems	Rapid muscle hypertrophy/Inelastic fascia

PACS is more prevalent in males, particularly adolescent males, and is associated with a higher incidence of high-energy traumatic injuries in patients with larger muscle mass.^1,20,22^ A critical driver of adolescent risk is the “Altered Ratio” theory.^1^ In adolescent males, the rapid growth of muscle mass often exceeds the capacity of the noncompliant, inelastic fascial envelope to expand. This precarious ratio makes the adolescent limb susceptible to critical pressure elevations following moderate trauma or exercise.^1,22,23^ Consequently, high-energy mechanisms, most notably pedestrian versus vehicle and motorcycle accidents, are the primary cause of PACS in modern trauma centres. This has replaced the historical risks associated with skin or skeletal traction and early Spica casting, which can contribute to compartment syndrome by mechanically increasing pressure within the muscle compartments and restricting the limb’s ability to expand during post-fracture swelling. Although both are standard non-operative treatments, primarily for paediatric femur fractures, improper technique or tight application can directly affect local haemodynamics.^8,11^ For example, femur fractures can be treated with a Spica cast or surgical fixation, such as flexible nailing or plate fixation. These methods eliminate the need for skin traction and long casting periods.^8,17^

**Figure 1.**
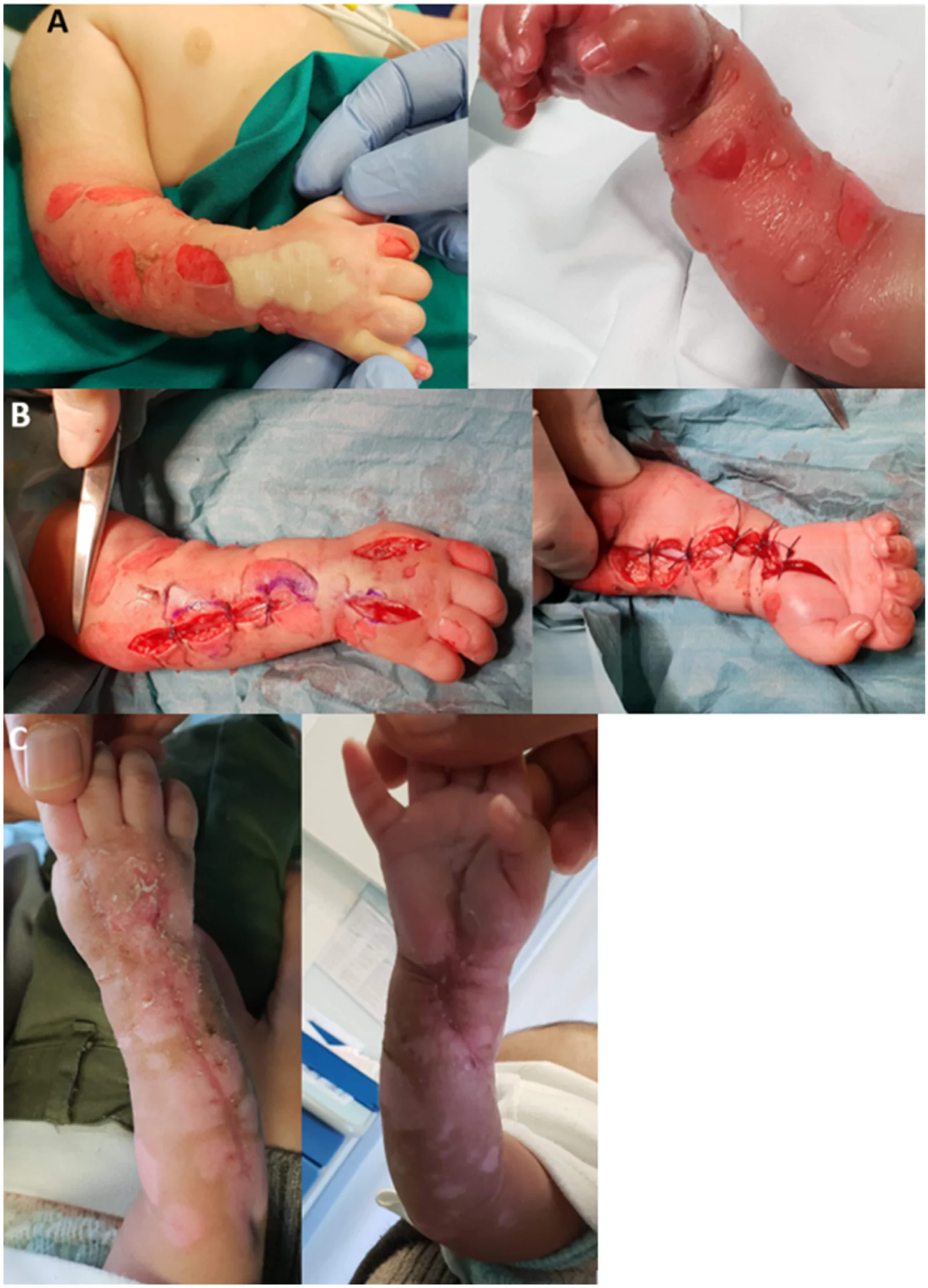
A 3-week-old neonate admitted for neonatal respiratory distress syndrome, with an intravenous line in the forearm secured using splints and bandages. Clinical appearance immediately before (A) and after (B) fasciotomy and one month after skin closure (C).

Today, the most common PACS-related fractures are forearm and tibial shaft fractures. The risk of supracondylar humerus fractures has decreased due to modern treatment methods.^2,17,18^ Approximately 40% of PACS are due to tibial shaft fractures.^1,20^ Although these fractures remain the predominant cause, modern practice must also account for the broader epidemiological spectrum of the disease, which is often more subtle. Providers must be vigilant for “incipient” and atypical presentations, such as neonatal limb compartment syndrome. This exceedingly rare condition presents at birth or during the immediate postpartum period. It primarily affects the upper extremities and is classically indicated by a “sentinel skin lesion,” such as blistering or necrosis, rather than by a traumatic event (Figure 1).

While the vast majority (up to 85%) of PACS cases are caused by traumatic fractures,^3^ there is a growing recognition of NFACS that pose unique diagnostic challenges and have specific epidemiological profiles.^1,9^

Vascular insults represent the most frequent etiology of PACS following fractures.^1^ In one study focusing exclusively on NFACS in children, vascular issues were the single most common cause, accounting for 28% of cases.^9^ The diverse etiologies within this category include intravenous fluid infiltration, arterial or intraosseous cannulation, deep vein thrombosis, spontaneous bleeding (e.g., from anticoagulation or hemophilia), and complications related to extracorporeal membrane oxygenation.^1^ The epidemiological risk profile for these vascular events contrasts with trauma-induced PACS; whereas trauma predominantly affects older adolescents, PACS resulting from vascular insults and infections occurs more frequently in younger children under 10 years of age.^1^ Furthermore, vascular-related PACS often presents atypically. These patients may not exhibit classic severe pain, but instead demonstrate high rates of poor perfusion (64%) and motor deficits (36%) upon initial presentation.^1,9^ Postischemic tissue is highly susceptible to slight increases in interstitial pressure. Vascular injuries can quickly lead to muscle ischemia and necrosis. Damage to blood vessels can cause bleeding or severe swelling, which can cut off blood flow. Ultimately, this warrants a lower threshold for surgical decompression. The causes and mechanisms fall into three categories:

*Vascular injury:* Direct tearing or trauma to an artery or vein can create a large, hidden hematoma inside the muscle.

*Ischemia-reperfusion:* When blood flow is blocked (ischemia) and then restored (revascularization), the sudden rush of oxygen can cause severe cellular swelling and fluid leakage.

*Tight space:* Since muscle fascia is essentially made of connective tissue that cannot stretch, internal pressure quickly builds up and compresses small blood vessels.^24,25^

**Figure 2.**
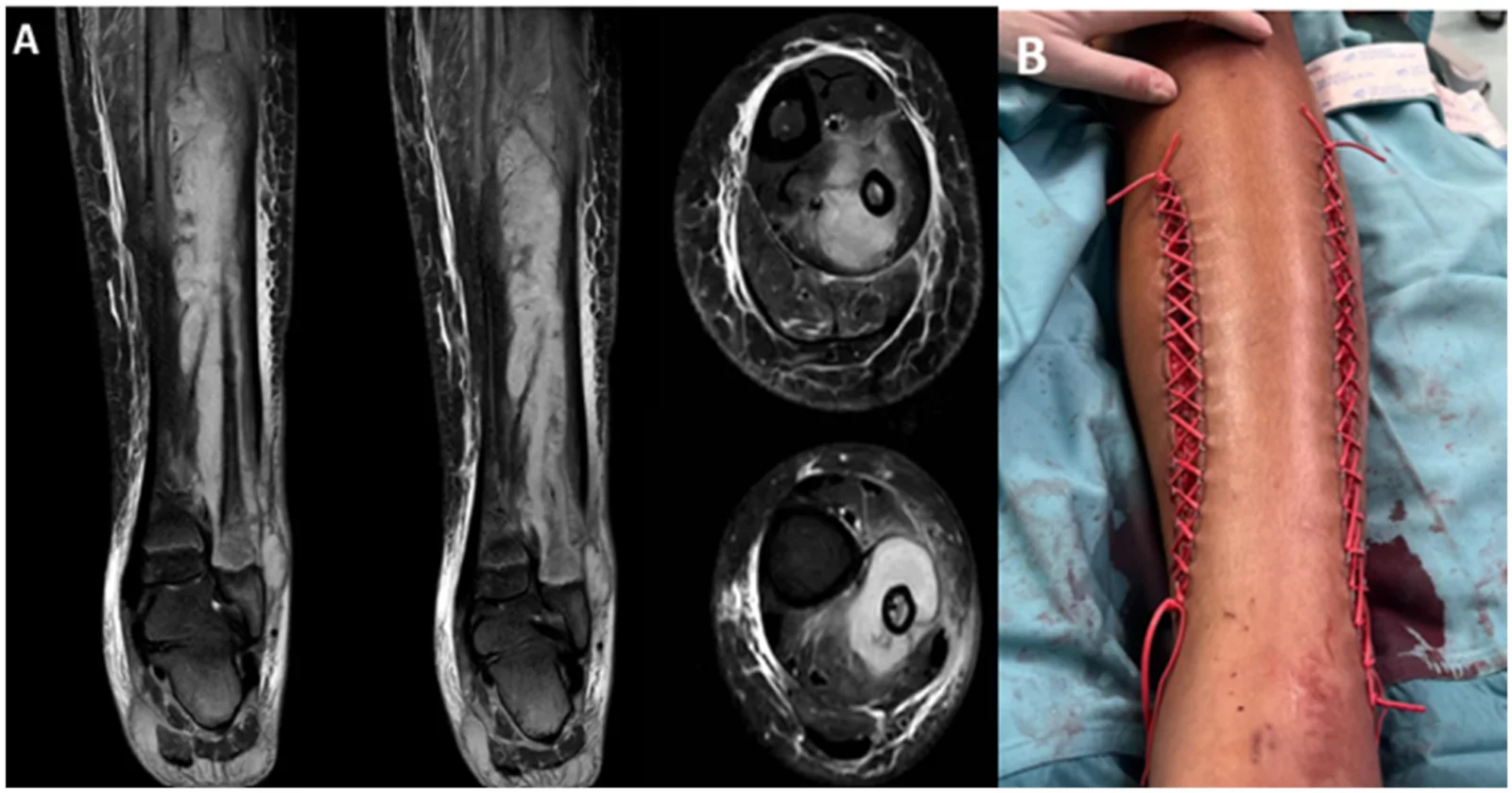
A 12-year-old patient with a history of a previous ankle sprain. MRI demonstrates peroneal osteomyelitis with a large abscess collection in the deep posterior compartment, accompanied by superficial and deep fasciitis (A). Post-operative outcome following compartment decompression and debridement via medial and lateral leg incisions, with skin closure achieved using the shoelace technique (B).

Infections are a rare atraumatic cause of PACS, representing about 10% of non-fracture cases.^9^ The epidemiology of infection-related PACS differs significantly from trauma-induced cases: while traumatic PACS predominantly affects older adolescents, cases arising from infections occur most frequently in younger children under 10 years of age.^1^ In studies evaluating NFACS in childrern, patients with infectious etiologies are often the youngest demographic, with one cohort averaging just 4.2 years of age.^9^ Furthermore, in a specific study analyzing infants and toddlers under 3 years old, infections accounted for 27% of all compartment syndrome cases.^15^ Infection-related compartment syndrome is primarily driven by Gram-positive organisms, most notably Methicillin-resistant *Staphylococcus aureus*, Methicillin-susceptible *Staphylococcus aureus*, and *Streptococcus pneumoniae*^15^ (Figure 2).

Although rare, bites from animals, insects, and snakes are documented causes of PACS.^17,26^ Children are particularly vulnerable to severe complications from snakebites because their smaller body size and lower total blood dilution volume make the venom more toxic.^27^ Beyond venomous snakes, PACS has also been reported following dog bites, spider bites, and flying insect bites.^17,26^

Improper or prolonged intraoperative limb positioning is a known iatrogenic cause of PACS. The greatest risk occurs during prolonged abdominal, pelvic, or urologic surgeries where the patient is placed in the dorsal lithotomy position.^1,3,9^

## Diagnostic evolution: The shift from the “5 Ps” to the “3 As”

The traditional diagnostic paradigm, based on the “classic five Ps” (pain, pallor, paresthesia, paralysis, and pulselessness), is an inadequate safeguard for children. These signs often represent late-stage ischemia and irreversible myonecrosis rather than early indicators and typically appear only after irreversible ischemic damage has occurred.^2,17,28^ Furthermore, preverbal or agitated children cannot provide the necessary subjective sensory feedback.^2,7,17^

Contemporary practice has shifted toward the “3 As”: anxiety, agitation, and analgesia requirements.^7^

*Anxiety:* the child exhibits increasing unexplained apprehension and restlessness.

*Agitation:* the child becomes inconsolable, uncooperative, and severely distressed, displaying a demeanour that is disproportionate to their injury.

*Analgesic requirements:* the child demonstrates an increasing need for pain medication, specifically higher doses or more frequent administration of opioids, which ultimately fail to provide adequate relief. Research by Bae et al. revealed that an increasing analgesic requirement is often the first sign of PACS, occurring several hours before neurovascular changes and uncontrolled pain.^2,7,10^

### Temporal safeguards

**Figure 3.**
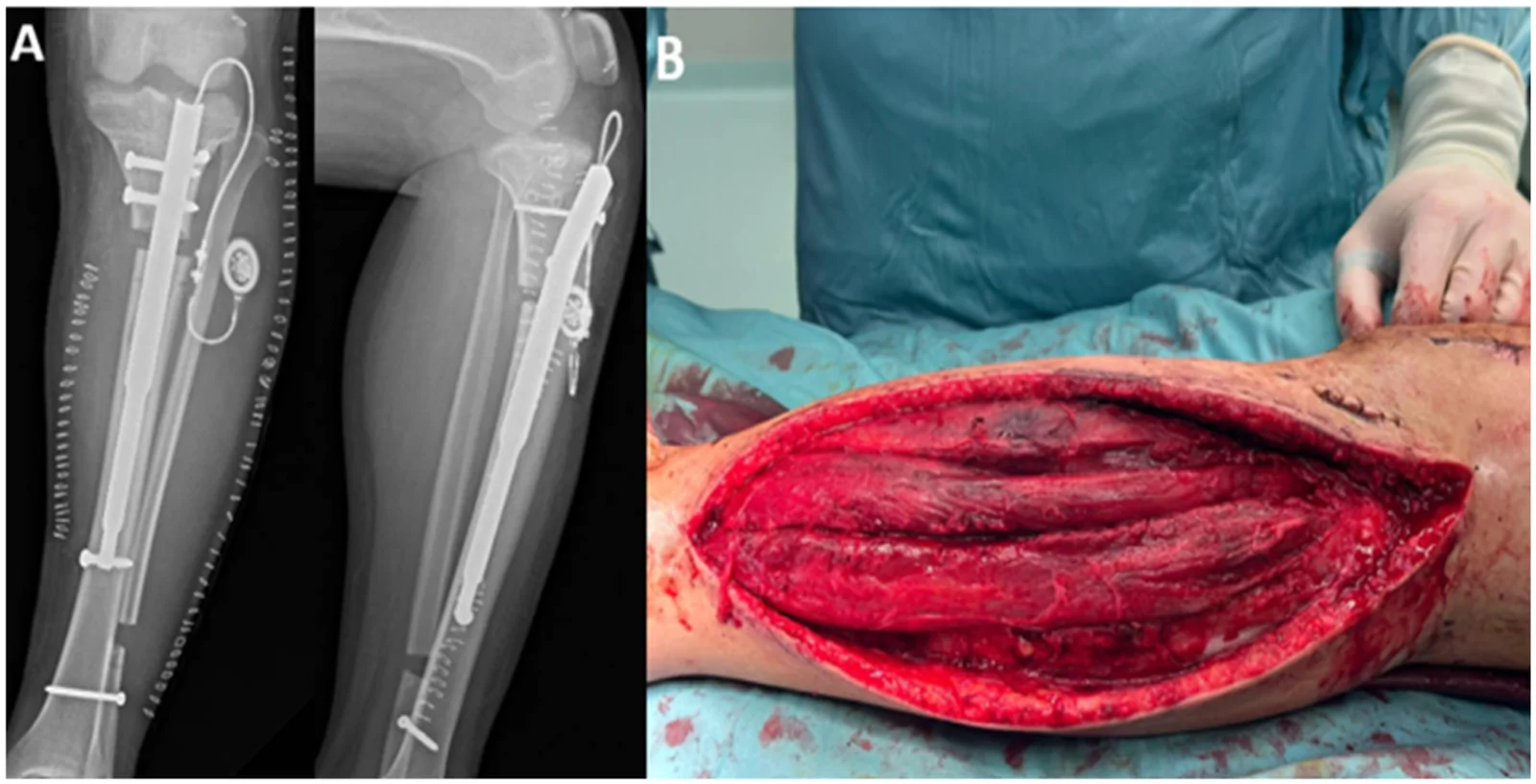
A 13-year-old female patient with lower limb length discrepancy secondary to a previous leg malignancy, who underwent limb lengthening with a magnetic intramedullary nail (A), complicated by compartment syndrome on post-operative day 2 (intraoperative view of the leg fasciotomy; B).

According to Bae et al., “analgesia demand,” or an increase in the frequency or dosage of narcotics (PCA or nurse-administered), is the most sensitive early indicator. This requirement has been shown to precede neurovascular signs by an average of 7.3 hours.^17^ It is crucial to understand this temporal window. Lin et al. reported a median injury-to-fasciotomy time of 20 hours,^10^ and Livingston et al. noted that NFACS experience a median delay of 48 hours.^9^ Recognizing the “3 As” is critical for safeguarding against these diagnostic blind spots (Figure 3).

In adults, delayed presentation of ACS is usually considered a contraindication for decompressive fasciotomy because the procedure performed late is more likely to result in severe infection and wound-healing complications, which outweigh the benefits of preventing irreversible nerve and muscle necrosis.^8,10^ However, according to contemporary orthopedic literature, pediatric ACS is considered a distinct clinical entity. Therefore, the traditional adult approach of withholding late fasciotomies should not be applied to children.^10^

The rationale for this divergence in management is multifactorial. First, pediatric patients have an good capacity for tissue healing and nerve regeneration, which allows for substantial functional recovery even after prolonged ischemic intervals. Additionally, the pathophysiological timeline of ACS appears to differ in children. The time between initial injury and peak compartment pressures is significantly longer in children than in adults, which expands the window of tissue viability.^8,10^

Robust clinical data supports an aggressive surgical approach in the pediatric population. Multiple case series report excellent functional outcomes and a notable absence of postoperative infections in children who underwent fasciotomies between 72 and 119 hours after their initial injuries.^8,10,15^ Consequently, while early recognition and intervention are the ultimate goals, surgeons are advised to proceed with decompressive fasciotomy in pediatric ACS patients with delayed presentations, as children consistently demonstrate superior tolerance to late surgical decompression without the devastating morbidities typically observed in adults.^1,8,10^

### Analgesia demand

Pain management in the context of acute compartment syndrome presents distinct challenges in children compared to adults, primarily because pain assessment in young, frightened, or non-verbal pediatric patients is inherently difficult.^7,17,29^ Adults may report excessive pain, while it is more difficult to assess children’s pain. Pain medication is a sensitive indicator of ACS, often preceding classic symptoms by hours. Balancing pain relief and risk is challenging, especially in children. Dense neuraxial anesthesia and opioid-based patient-controlled analgesia are discouraged in high-risk patients because they can mask the breakthrough pain of compartment syndrome as it develops. Contemporary approaches recommend using low-concentration, site-specific nerve blocks for pain management and diagnosis.^1,6,7,9,17,29^

It is difficult, if not impossible, to perform invasive compartment pressure measurements on an awake, injured child. Young children, especially infants and toddlers, are usually too anxious to cooperate with having needles inserted into their injured limbs. This often requires conscious sedation or general anesthesia. However, if a child’s condition is serious enough to require surgery, the surgeon will perform a decompressive fasciotomy.^8,11,15,29^

Contemporary pediatric orthopedic practice relies on clinical assessment, such as the “3 A’s,” rather than absolute pressure numbers for diagnosing PACS.^18^ Invasive pressure measurements are not routine for young children, but they are used in specific high-risk scenarios or as a confirmatory measure in the operating room after anesthesia.^18,30^

Managing a fasciotomy wound in a pediatric patient typically necessitates repeated general anesthesia. Following initial decompressive surgery, the standard of care requires the child to return to the operating room for serial washouts, tissue reassessment, debridement, and incremental wound closure.^3,5^ Because dressing changes and wound manipulations are distressing and painful for young patients, these sequential procedures are routinely performed under general anesthesia.^18^

Modern dermatotraction devices gradually pull the edges of the skin together at the bedside, eliminating the need for an additional trip to the operating room. However, these specialized mechanical devices are not widely used on children because many cannot tolerate the procedure at the bedside.^18^ Consequently, reliance on general anesthesia is a recognized short-term morbidity of fasciotomy wound management in children. It contributes to prolonged hospital stays and delayed rehabilitation while the clinical team works to close the wounds successfully.^4,18^

### Diagnostic warning checklist

According to the current evidence, the following diagnostic warning checklist provides a structured guide to help interpret four clinical signs that suggest compartment syndrome:^3,7,10,17,18,30^

1. Uncontrolled pain is defined as pain that is out of proportion to the injury or refractory to escalating narcotic doses.^17,30^
2. Pain on passive stretch, which corresponds to exquisite discomfort during passive extension of the involved compartment’s musculature.^3,17^
3. Agitation and anxiety that increase over time and cannot be explained by environmental factors;^7,10,18^
4. Palpable tenseness, which is identified upon physical palpation of the affected limb.^3,30^

## Intracompartmental pressure monitoring and thresholds

Objective ICP monitoring must be sought when clinical signs are equivocal, particularly in obtunded, sedated, or very young patients.^1,6,7,10,18^ However, the values obtained during the clinical assessment must be interpreted in the context of the patient’s age. The physiological characteristics of children differ from those of adults and from those of children of different ages. This affects how these values are interpreted:

- *Resting pressures.* Children have higher normal resting pressures (13.3-16.6 mmHg) than adults (5.2-9.7 mmHg).^1,7,8^
- *Absolute thresholds.* The difference in baseline resting compartment pressure between children and adults is clinically significant because standard fasciotomy thresholds, such as an absolute pressure greater than 30 mmHg or a perfusion gradient less than 30 mmHg, are based on adult models.^7,8,28^ Applying these adult cutoffs to children, who naturally operate at higher baseline compartment pressures and lower systemic blood pressures, is unreliable and risks unnecessary surgical interventions.^1,7,8,28^
- *Delta pressure* (ΔP) is calculated as diastolic blood pressure minus ICP. A ΔP <30 mmHg is the standard threshold for decompression, though some evidence suggests that children may remain asymptomatic until ΔP <20 mmHg.^1,7,28^
- *Vascular thresholds.* In cases of vascular injury, post-ischemic tissues undergo hypoxic metabolic changes at higher pressure differentials. For these patients, a more aggressive threshold (ΔP <40 mmHg) for fasciotomy is warranted to preserve compromised tissue.^1,30^

### Modern diagnostic adjuncts

Near-infrared spectroscopy (NIRS) is a non-invasive technology that measures tissue oxygenation by detecting the different light absorption properties of oxygenated and deoxygenated hemoglobin. It can sample tissue up to 3 cm below the skin’s surface, providing continuous, real-time tissue oximetry.^6,7,28^

NIRS is a promising diagnostic tool for PACS, but it’s not the gold standard for diagnosing it in children. Although it has been evaluated in adults, researchers have not established universally accepted or standardized cutoff thresholds that reliably indicate critical tissue ischemia in growing individuals.^6–8,18,28^

NIRS has its advantages and disadvantages. Among its advantages is that it is non-invasive and avoids the pain and agitation associated with inserting a needle manometer into an awake child. NIRS also allows for continuous monitoring, providing an ongoing, real-time assessment of tissue oxygenation rather than a single pressure reading at a specific point in time. Additionally, NIRS can assess oxygenation in muscle tissue up to 3 cm below the skin surface (deep tissue sampling).^6–8,15,28^

However, the technology has some limitations. Notably, there is a lack of standard thresholds and defined cut-off values for definitively diagnosing significant tissue ischemia.^7,18^ Furthermore, variations in skin pigmentation and subcutaneous fat thickness can negatively impact accuracy.^15^ Further study is needed before the technology can replace clinical evaluations or invasive manometry and be safely and routinely integrated into everyday pediatric clinical practice^15,28^

## Traumatic PACS: Fracture-specific risk profiles

According to Villarreal et al. and Shore et al., age over 14 years, higher body mass index (BMI), and comminuted or segmental fracture patterns are risk factors for PACS secondary to trauma.^16,23^ Tibial shaft fractures are the most common cause of post-traumatic PACS, but other fracture patterns can also cause it in children.

### Tibial shaft fractures

Tibial shaft fractures are the primary cause of traumatic PACS, accounting for over 40% of cases, although other fracture patterns can also cause PACS in children.^1,8,20,31^ The treating surgeon should pay close attention to any clinical picture compatible with compartment syndrome.^1,8,20^

### Extensor retinaculum syndrome

Extensor retinaculum syndrome is an acute entrapment neuropathy predominantly affecting children aged 10 to 15 following displaced distal tibial physeal fractures.^32^

Anterior displacement of the fractured tibia compresses the underlying structures within the rigid superior extensor retinaculum, causing localized ischemia that mimics a compartment syndrome.^32^

Anatomical studies confirm that the extensor hallucis longus muscle is particularly vulnerable to this localized compression because a significant proportion of its muscle fibers extend directly beneath the superior extensor retinaculum and it receives a relatively sparse arterial blood supply.^33^

Patients classically present with disproportionate ankle pain, numbness in the great toe web space (indicating deep peroneal nerve compression), toe extension weakness, and intense pain upon passive toe flexion.^32^

Diagnostically, tissue pressures are increased (>40 mm Hg) strictly beneath the retinaculum, while pressures in the anterior leg compartment remain normal.^32^

### Tibial tubercle avulsion fractures

Bergen et al. reported a 0% acute compartment syndrome rate in a recent cohort with stable Ogden types I–III tibial tubercle avulsion fractures (TTAF), suggesting that these fractures can be managed with same-day discharge. However, a high level of caution is required for Ogden types IV and V TTAF because extension to the posterior metaphysis increases the risk of injury to the anterior tibial recurrent artery.^34,35^

### Femur fractures

The transition to immediate Spica casting or surgical fixation with elastic nailing has largely mitigated the historical risk of PACS in pediatric patients with femur fractures managed with skin and skeletal traction.^1,7,17^

Skin traction uses strips or boots wrapped with elastic bandages to pull the leg longitudinally. It can cause compartment syndrome in several ways:

*The bandages are wrapped too tightly*: As the leg swells from initial trauma, these tight bandages act as a tourniquet, trapping fluid and elevating pressure.

*Excessive traction weight*: Pulling forces that are disproportionate to a child’s weight overstretches the fascial envelopes. This narrows the muscle compartments, increasing internal pressures.

*Arterial spasms and ischemia:* Limb elevation can trigger arterial spasms, restricting blood flow.

*Uninjured limb vulnerability*: In setups like Bryant’s traction (where both legs are suspended vertically), compartment syndrome can develop in the uninjured leg due to sustained elevation and tight bandaging.^1,17,36,37^

### Supracondylar humerus fractures

Supracondylar humerus fractures are the leading cause of upper extremity compartment syndrome in children.^17,31^ The risk increases with severe displacement, associated vascular injury, or treatment involving closed reduction and casting with elbow flexion exceeding 90 degrees.^7,17,18^ This treatment artificially reduces compartment volume and compresses the local vasculature. Notably, while the baseline risk for isolated upper extremity fractures is very low, research demonstrates that when a displaced extension supracondylar humerus fracture is combined with a displaced ipsilateral forearm fracture, the absolute risk of developing compartment syndrome raises to 33%.^38^ Because exactly one-third of pediatric patients with this specific combination of displaced fractures will develop the condition, a floating elbow places the child at increased risk, necessitating mandatory inpatient monitoring and close perioperative observation.^7,18^

### Forearm fractures

Acute compartment syndrome is rare in pediatric forearm fractures. It most commonly affects boys between the ages of 10 and 14 and is often the result of high-energy trauma, open fractures, or extensive surgical manipulation.^3,5–7,17,18^

### Crush injuries of the foot

Crush injuries are the most frequent cause of ACS in the pediatric foot.^39,40^ Diagnosing this condition is challenging due to communication barriers with young children, inconclusive radiographs resulting from skeletal immaturity, and the fact that classic neurovascular signs like pulselessness or paralysis are unreliable and typically absent during the initial presentation.^39,40^ Clinicians must maintain a high index of suspicion when a patient presents with marked swelling, bruising, and severe pain that is disproportionate to the injury and exacerbated by passive toe movement.^39,40^

If the condition is suspected, the diagnosis should be confirmed using multiple ICP pressure measurements, specifically targeting the central and interosseous compartments because they are the most sensitive indicators.^39,40^

## Non-Fracture Acute Compartment Syndrome

NFACS is characterized by a significant diagnostic “blind spot,” with median delays of 48 hours resulting in a high rate of myonecrosis (54%).^9,18^

### Extravasation, casts, and crush injuries in child abuse

Atraumatic or non-fracture etiologies often suffer from delayed diagnosis and carry a high rate of subsequent myonecrosis.

The poor recovery rate (56%) in NFACS compared to fracture-related ACS (>90%) is primarily attributed to the lack of “heightened awareness” clinicians maintain for skeletal injuries.^9,17^

The etiologies of NFACS can be categorized as follows:

1) *Trauma:* Blunt force or crush injuries without fracture;^9,15^
2) *Infectious*: Deep spontaneous infections;^15^
3) *Postoperative:* Prolonged positioning (e.g., dorsal lithotomy) or regional anesthesia that masks symptoms;^1,9^
4) *Exertional:* Exertional Compartment Syndrome, is a rare, limb-threatening condition caused by acutely elevated compartment pressure triggered solely by physical exertion, without any discrete traumatic event. Because it mimics more benign conditions, it is frequently misdiagnosed by physicians and athletic trainers as a muscle strain or shin splints, leading to dangerous treatment delays;^1,41^
5) *Extravasation of intravenous fluids:* Iatrogenic vascular injuries are a leading cause of non-fracture PACS. The pediatric population is distinctly vulnerable to intravenous infiltration injuries due to the fragility of veins in children and the increased velocity of fluid administered through smaller catheter tips;^9,15,42,43^
6) *Tight casts:* Any external force that decreases the volume capacity of a compartment can cause ischemia. Restrictive, circumferential dressings and tight casts can create compression that compromises microcirculation. Similarly, immobilization techniques, such as 90/90 Spica casting for femur fractures, can also cause ischemia;^1,7,43^
7) *“Crush” Injuries and Non-Accidental Trauma:* Providers must maintain a high level of suspicion for severe soft-tissue crush injuries, especially in cases of child abuse. These mechanisms cause massive, rapidly evolving interstitial edema. Since caregivers often provide vague, misleading, or absent histories for these injuries, recognizing disproportionate swelling and distress is critical for preventing irreversible limb loss and other systemic injuries.^8,15^

### The pediatric reflex: High-energy mechanisms in a flexible skeleton

To understand why children develop ACS, it is necessary to examine their unique musculoskeletal physiology. Because the pediatric skeleton is highly elastic and resilient yet porous, children with healthy bones require a greater amount of energy to sustain a fracture than adults do.^44,45^ Consequently, when a child experiences a high-energy impact (e.g., a motor vehicle collision), a significant amount of force is absorbed by the surrounding soft tissue per unit area.^22^ Children, especially adolescent males, have rapidly growing muscle masses that are tightly encased within thicker, tighter, and relatively inelastic fascial envelopes.^1,16,20,22^

This anatomical mismatch limits the compartment’s ability to expand. When the trauma’s massive energy induces acute interstitial bleeding and edema, the unyielding pediatric fascia causes ICP to spike rapidly, creating the perfect storm for devastating ischemia.^1,3,11,15,22^

## Management: Decompression and wound care

The standard of care for established PACS is emergent, wide-incision decompressive fasciotomy.^17,18^ The mantra “time is tissue” and “if in doubt, do a fasciotomy” remains the governing principle.^11,28^

The consequences of a missed diagnosis (such as Volkmann’s contracture and limb loss) are devastating; the threshold for surgical intervention in children is often lower than in adults.^1,5,18,20^ Consequently, “prophylactic” fasciotomies are relatively common in high-risk scenarios, such as during proximal tibial osteotomies or when clinical suspicion remains high despite equivocal objective data.^1,17,18,28,46^

Prophylactic fasciotomy is recommended for patients at risk of developing ACS, particularly following prolonged ischemia, reperfusion injury, high-energy trauma, or fractures. The procedure is performed before symptoms appear. The surgical technique is chosen based on the clinical setting. Open long-incision fasciotomy is preferred for acute traumatic conditions, while limited-incision approaches are used for elective procedures.

For acute trauma, such as tibial fractures (about 45% of ACS cases), the gold standard is complete decompression through full-length skin (10 to 15 cm) and fascial incisions. This approach releases all compartments, prevents constrictive skin after fascial opening, and facilitates accurate identification of intermuscular septa. It also minimizes the risk of incomplete decompression of the anterior or deep posterior compartments. In contrast, smaller 4 to 5 cm incisions can be used for elective procedures, such as tibial osteotomies or chronic compartment syndromes, where severe edema and ischemia-reperfusion injury are not anticipated. Decompression in these cases is achieved by extending the fascial release proximally and distally through the limited incision using blunt-tipped instruments. However, limited or percutaneous fasciotomy techniques are generally contraindicated in acute lower extremity ACS because restricted exposure may result in incomplete compartment release. This can lead to persistent intracompartmental hypertension, muscle necrosis, increased morbidity, and potentially, mortality. Furthermore, blind fascial division is technically challenging because it is difficult to maintain fascial tension, and limited visualization increases the risk of iatrogenic injury to the superficial peroneal nerve. While a limited-incision technique is a documented, less invasive alternative in elective tibial procedures, surgeons must ensure adequate proximal and distal fascial release. Any postoperative evidence of acute compartment syndrome should prompt immediate conversion to a formal full-length open fasciotomy.^1,17,18,20,22,28,46^

### Non-surgical management

When PACS is suspected, the initial non-operative management must be rapid and include the following steps:

1) Remove all circumferential dressings, and split or bivalve casts down to the skin. Cutting and opening a plaster cast can reduce external cast pressure by 40% to 60%, while cutting the underlying restrictive bandages can reduce pressure by up to 80%.^22,37^
2) The affected limb should be kept at heart level and not elevated. Traction on the limb should also be avoided. Elevating the limb decreases the arteriovenous pressure gradient, which can further compromise critical microcirculation and worsen tissue ischemia, unlike general edema^1,18^

### The surgical protocol

The gold standard for treating PACS is a fasciotomy performed in a timely manner.^3,6,8,29^ Complete decompression is essential. After fasciotomy, surgeons must evaluate muscle viability using the “four Cs”: color, consistency, contractility, and capacity to bleed.^18^

For the leg, a four-compartment release is usually performed using two long incisions: an anterolateral incision and a posteromedial incision. Alternatively, all four compartments can be successfully decompressed using a single-incision technique, which involves a long lateral incision extending from just distal to the fibular head down to the lateral malleolus.^3,11,18^

The thigh is divided into three distinct compartments: the anterior, posterior, and adductor compartments. Decompression of both the anterior and posterior compartments can usually be achieved through a single lateral incision. The third compartment, the adductor compartment, is rarely involved,^18^ Tarsal tunnel release is often part of the urgent surgical decompression required for tibial nerve.^39,47^

PACS in the upper extremity is considered extremely rare.^6,18^ When decompression is necessary, the anterior and posterior compartments are typically released using lateral incisions on both sides.^6^

For the forearm, volar and dorsal releases are standard, combining the opening of the carpal tunnel to prevent median nerve damage.^3,18,24^ An S-shaped or curvilinear incision often suffices to decompress all volar compartments, though a supplementary dorsal incision may be. Necessary if pressures remain elevated in the dorsal or mobile wad compartments.^6,18,31,48^

For the hand, decompression is typically achieved via two dorsal incisions over the second and fourth metacarpals.^6,18^ The same applies to the foot, where decompression is achieved via one or two dorsal incisions over the second and fourth metatarsals.^18^

### Wound management

The next important step after fasciotomy is wound management.

Delayed primary closure is typically attempted after 48–72 hours and is successful in a large proportion of cases.^4,5,18^ Vacuum-assisted closure (VAC) therapy is used after fasciotomy to reduce swelling and promote granulation tissue growth.^4,18,37^ After VAC treatment, direct closure may be possible.

In 12%-21% of cases where edema precludes primary closure, split-thickness skin grafting or tissue expansion is necessary.^1,9^

Fasciotomy wound closure remains challenging, requiring a balance between delayed primary closure and minimizing complications. The *shoelace technique* is one of the most widely used methods because it is simple, safe, and inexpensive. Staples are placed along the wound edges and connected with a vessel loop in a crisscross pattern, which is tightened every 48 hours as edema resolves (Figure 2(b)). Several modifications have been described. The technique is particularly useful in resource-limited settings and often avoids the need for skin grafting, although excessive tension may cause skin ischemia, necrosis, or staple detachment.^49–51^

A second approach, *dynamic dermatotraction,* uses mechanical devices to exploit the viscoelastic properties of the skin (the elongation of skin under constant load over time).and promote gradual wound closure.^49,51–53^ The third major strategy is *vacuum-assisted closure (VAC) or negative pressure wound therapy (NPWT)*, which uses foam dressings connected to a vacuum pump. NPWT reduces edema, removes wound fluid, stimulates angiogenesis, and may decrease bacterial burden. Although it is associated with higher costs and a greater likelihood of requiring skin grafting, it is considered the safest option for high-risk patients.^51–53^

Secondary intention healing, in which the wound is left to close on its own, has largely been abandoned. It is associated with high rates of infection, prolonged hospitalization, muscle necrosis, and excessive scarring. It is now only used as a last resort when primary closure fails due to infection.^54–58^

A meta-analysis of 23 studies including 606 patients revealed that dynamic dermatotraction had the highest closure success rate (92.7%), followed by gradual suture approximation (92.4%). VAC achieved a success rate of 78.1%. However, dynamic dermatotraction and gradual suture approximation had the highest complication rates (18.4% and 14.83%, respectively), while VAC had the lowest rate (2.49%). These results demonstrate the importance of balance between maximizing primary closure and minimizing complications.^1,9,37,49–53^

Successful closure depends on allowing edema to subside before applying tension. Premature or excessive tension can lead to skin necrosis, delayed healing, recurrent compartment syndrome, infection, and hypertrophic scarring. Consequently, there is no definitive gold-standard technique, and treatment should be tailored to the individual patient. Overall, simple suture approximation methods, particularly the shoelace technique, remain among the most commonly used and effective options.

### The pediatric recovery paradox

Clinicians should be conservative with initial debridement in children. Pediatric muscle recovery is more robust than in adults; tissue that appears dusky or non-contractile on initial inspection often demonstrates reversible myonecrosis and recovers viability during subsequent serial washouts (average of 3 surgeries).^1,3,18,41^

## Functional outcomes and long-term recovery

The long-term recovery and functional outcomes of pediatric compartment syndrome largely depend on the underlying etiology and the timeliness of the surgical intervention.^59^ Despite the potential for devastating complications such as irreversible muscle necrosis, Volkmann’s contracture, and permanent nerve damage, pediatric patients generally exhibit a remarkably robust capacity for tissue and nerve recovery.^1,59^ Consequently, children often achieve better functional outcomes than adults, even in cases where surgical decompression is substantially delayed.^3,10,18^

1. *Acute Compartment Syndrome* has a relatively good prognosis due to the resilience of pediatric tissue, and the majority of children achieve excellent functional outcomes and full recovery. They often return to their pre-injury activity levels.^8,60^ While long-term complications are uncommon, diagnostic delays can result in persistent muscle weakness, stiffness, or devastating sequelae like Volkmann’s contracture.^8,59,60^
2. *Nonfracture Acute Compartment Syndrome (NFACS)* has a significantly worse prognosis than fracture-related ACS due to its atypical presentation, with 54% of patients demonstrating myonecrosis at the time of surgery.^9,18^ Only about half of children with NFACS achieve a complete functional recovery, while the rest often suffer from persistent pain, or neurological and functional deficits^9^ (Table 2).
3. *Exertional Compartment Syndrome* is triggered by physical exertion and normalizes with rest, meaning it rarely causes the limb-threatening tissue necrosis or permanent nerve injury seen in acute presentations. Patients are safely and successfully managed with elective fasciotomy, allowing for an excellent long-term functional recovery.^41^(Table 3).

**Table 2.** Clinical characteristics and outcomes of acute pediatric compartment syndrome.

Condition or fracture pattern	Prevalence/Incidence	Key diagnostic indicators	Treatment/Management protocols	Reported outcomes/Complications	Source
Pediatric fractures (tibia, fibula, distal radius, ulna, supracondylar humerus), crush injuries, and sports trauma.	75% associated with fractures; 0.1% to 3.3% incidence in long bone fractures; higher risk in males and older adolescents.	Pain out of proportion (83-88%), 3 A’s (Anxiety, Agitation, Analgesia requirement), tense swelling, and pain with passive stretch.	Immediate surgical decompression (fasciotomy) of all affected compartments; fracture fixation (ORIF, pins, or flexible nails); maintain normal blood pressure.	90% achieve full recovery if treated early; complications include muscle necrosis, Volkmann contracture, nerve palsy, and limb loss.	^5–8,11,15,19,22,25,28,30,35,40,42–44^
Supracondylar humerus fractures (SCHF), specifically Gartland type III.	0.1% to 0.3% incidence; 19% nerve injury rate in type III fractures.	Weak/absent radial pulse, cold extremity, sensory loss (median, radial, or ulnar nerves), and muscle tenderness.	Closed reduction and percutaneous pinning (CRPP) or overhead skeletal traction; emergent arm/forearm fasciotomy if indicated.	90-94% satisfactory outcomes; risks include iatrogenic ulnar nerve injury and cubitus varus deformity.	^7,28,42,44^
Tibial tubercle avulsion fractures (TTAF).	0% to 1.35% prevalence; associated with damage to recurrent anterior tibial artery.	Ogden Type IV/V injuries (extension to posterior metaphysis); physical exam (5 P’s/3 A’s).	Surgical fixation (screws, K-wires) and hospital monitoring; prophylactic fasciotomy is common in literature.	Low prevalence of ACS but high risk of devastating sequelae if missed; hardware removal required in 6%.	^30,37^
Pediatric viper/snake bites (e.g., Vipera ammodytes).	4.2% (1 in 24 cases) over a 10-year period.	Hard oedema, ecchymosis, extreme pain on passive movement, and high CPK levels.	Emergency fasciotomy, antivenom administration, supportive care, and Vacuum-Assisted Closure (VAC) therapy.	Favourable progression with secondary suturing; high risk of tissue loss if untreated.	^26^
Neonatal Limb Compartment Syndrome (NLCS).	Rare; 86 cases reported (1980-2021).	Sentinel skin lesion (94% - blue plaque, eschar, blisters), cyanosis (89%), and flaccid paralysis.	Surgical decompression (32.6%) vs. nonsurgical management (68.6%).	16.6% sequelae if treated <24h vs. 81.3% if late; results in Volkmann contracture and bone abnormalities.	^34^
Acute Exertional Compartment Syndrome (AECS).	Rare; 7 cases in a 15-year study.	Older adolescent male athletes; pain out of proportion after exercise; high pressures (mean 91 mmHg).	Emergent surgical decompression (primarily anterior and lateral compartments).	57% initial missed diagnosis rate; 57% muscle necrosis; persistent foot drop in delayed cases.	^32^

**Table 3.** Clinical characteristics and outcomes of chronic pediatric compartment syndrome.

Condition or fracture pattern	Prevalence/Incidence	Key diagnostic indicators	Treatment/Management protocols	Reported outcomes/Complications	Source
Volkmann’s Ischemic Contracture (VIC)/Neglected ACS.	37 patients identified over 3 years; more common in developing countries.	Irreversible tissue necrosis, fixed flexion contractures, claw hand, and muscle atrophy.	Excision of fibrosed muscle, neurolysis, tenolysis, Z-lengthening, and tendon transfers.	Reasonable improvement in range of motion; completely normal function is often impossible.	^13,45^
Chronic Exertional Compartment Syndrome (CECS).	Not statistically quantified in sources.	Exercise-induced pain and moderately elevated pressures that normalize with rest.	Elective fasciotomy.	Excellent long-term functional recovery; rarely causes permanent injury.	^32^

## Chronic exertional compartment syndrome

Chronic exertional compartment syndrome (CECS) is a debilitating but non-limb-threatening condition characterized by a reversible increase in pressure within a non-compliant, inelastic fascial compartment during physical activity.^61,62^ This elevated pressure compromises local tissue perfusion, leading to transient ischemia and neuromuscular dysfunction.^61,62^ Unlike acute compartment syndrome, which is a surgical emergency typically caused by trauma, CECS is predictably triggered by exertion and reliably resolves once the offending activity ceases.^61,62^ While CECS can occasionally occur in the upper extremities of rowers or motorcyclists, it predominantly affects the lower extremities.^61^ Specifically, the anterior and lateral compartments of the lower leg (the calf) are the most frequently involved anatomical zones.^63^

Although CECS has historically been studied in adult military personnel, it is increasingly recognized in the pediatric and adolescent populations.^61,64^ In this demographic, it most commonly affects adolescent female athletes (average age of 16) who participate in running-intensive sports such as track, soccer, and field hockey.^64^ The pathophysiology in adolescents is suspected to involve rapid muscle hypertrophy during growth that outpaces the expansion of the surrounding fascial envelope.^41^ Patients typically present with chronic, activity-induced lower leg pain, tightness, weakness, or numbness (such as foot drop) that worsens as exercise continues and dissipates within minutes to hours of resting.^62–64^

The diagnosis of CECS is often delayed because physical examinations at rest are typically unremarkable.^61^ It is essentially a clinical diagnosis supported by objective testing to rule out overlapping conditions like medial tibial stress syndrome (shin splints), stress fractures, or popliteal artery entrapment syndrome.^62,63^ The current gold standard for confirming CECS is intramuscular compartment pressure (IMCP) testing using the Pedowitz criteria, which involves measuring tissue pressures within the specific lower leg compartments at rest and at 1-minute and 5-minute intervals after an exercise treadmill protocol.^63,64^

However, these diagnostic criteria—originally established entirely in adult populations—present several profound clinical and practical limitations when applied to pediatric patients.^6,28,64,65^ First, children physiologically maintain significantly higher baseline resting compartment pressures than adults, with normal pediatric values averaging 13 to 16.6 mmHg compared to just 5.2 to 9.7 mmHg in adults.^1,6–8^ Applying the absolute adult-derived Pedowitz resting threshold of 15 mmHg to children therefore introduces an exceptionally high risk of false-positive diagnoses in asymptomatic young athletes.^28^ Second, because pediatric patients have lower baseline systemic blood pressures, they operate under narrower perfusion gradients (diastolic blood pressure minus compartment pressure), routinely tolerating small gradients (30 mmHg) without displaying any tissue ischemia.^28,29^ Third, absolute pre-operative pressure measurements and the number of positive Pedowitz criteria fail to correlate with or predict post-operative symptom relief, return-to-sport success, or patient satisfaction in younger cohorts.^64,65^ Fourth, obtaining these dynamic measurements requires an invasive, painful procedure that is poorly tolerated by anxious awake children; the resulting distress and muscle guarding can artificially spike tissue pressures, thereby compromising diagnostic reliability.^7,18^ Finally, the testing is highly sensitive to confounding technical variables and does not account for the prolonged pressure normalization time, which many pediatric investigators consider to be the most characteristic feature of the disease.^63,66^

Management of CECS should initially begin with a trial of nonoperative strategies. These include prolonged rest, physical therapy, and biomechanical interventions such as gait retraining.^63^ For example, transitioning a runner to a forefoot strike pattern can significantly reduce the biomechanical load on the anterior and lateral compartments of the lower leg.^61,63^ Additionally, ultrasound-guided botulinum toxin A (Botox) injections into the affected musculature have emerged as a promising conservative treatment to reduce exertional compartment pressures.^61,63^

For pediatric patients who fail conservative management and wish to return to high-level athletics, the definitive treatment is an elective surgical fasciotomy to release the tight fascial compartments.^63,64^ In children and adolescents, minimally invasive endoscopy-assisted compartment releases are highly favored to minimize surgical morbidity.^61^ Surgical intervention in the pediatric population is generally highly successful, yielding a return-to-sport rate of nearly 80%.^64^ However, families must be counseled on the risk of recurrence. Pediatric studies demonstrate an 18.8% recurrence rate requiring reoperation; notably, the odds of surgical failure and recurrence are 3.4 times higher when surgeons perform an isolated release of only the anterior and lateral compartments of the lower leg, compared to a comprehensive release of all four lower leg compartments (which also includes the superficial and deep posterior compartments).^64^

## Guidelines and action to take in clinical practice: A strategic clinical summary

Clinicians must maintain a heightened index of suspicion across all etiologies, utilizing the “three A’s” for early detection.^30,44^ In the neonatal population, diagnosis remains strictly clinical and necessitates the prompt recognition of sentinel skin lesions, such as blistering or focal necrosis, characteristically presenting on the forearm postpartum.^7,37,43^ Intracompartmental manometry serves strictly as a diagnostic adjunct for uncooperative or obtunded patients.^7,10,30^

Initial non-operative management mandates immediately removing circumferential dressings, bivalving casts,^17,22,30^ maintaining the limb at heart level to optimize the arteriovenous pressure gradient, and supporting hemodynamic normotension.^30^

The definitive standard of care remains an emergent, wide-incision decompressive fasciotomy across all affected compartments, strictly adhering to the maxim “if in doubt, do a fasciotomy”.^11,18,28^

Standard surgical approaches include a dual or single-lateral incision for the leg,^11,18^ a volar curvilinear incision for the forearm, and dual dorsal incisions for the hand and foot.^6^ Intraoperatively, surgeons must exercise extreme conservatism with primary muscle debridement due to the robust regenerative capacity of pediatric tissue.^1,6,18^

Post-decompression wounds must remain open and be managed with negative pressure wound therapy and serial washouts every 48 to 72 hours, optimizing the wound bed for eventual delayed primary closure.^1,3,4,18^

## Conclusions

The prognosis for PACS is generally excellent when treatment occurs within the critical ischemic window. Over 90% of children achieve full functional restoration within six months.^17^ However, the consequences of delay, especially in NFACS, are severe and can include persistent neurological deficits, myonecrosis, and the rare necessity of amputation.^9^

Conditions such as intravenous fluid infiltration, infection, tight casts, and neonatal compartment syndrome can be easily misdiagnosed.^15,43^ Preventing long-term disability depends entirely on the surgeon’s ability to prioritize the behavioral “3 As” over late-stage neurovascular changes.^7,10^

The surgical management of PACS highlights the remarkable healing capacity of the developing child. Unlike adults, children routinely achieve excellent functional recovery even when decompressive fasciotomy is significantly delayed (e.g., 24 to 72 hours post-injury).^2,8,15^ Furthermore, pediatric patients have a high success rate with delayed primary closure over multiple serial debridements, which minimizes the need for cosmetically inferior skin grafting.^1,4,6^

Diagnosing pediatric CECS remains challenging, relying heavily on clinical suspicion and dynamic ICP testing.^63,64,66^ Epidemiologically, it most commonly affects adolescent female runners in the lower extremities,^64^ and competitive motorcyclists in the upper extremities.^56^Although conservative measures like gait retraining and botulinum toxin A injections show promise,^63^ decompressive fasciotomy remains the definitive treatment, allowing approximately 80% of young athletes to successfully return to sports.^64,66^ However, clinicians must be aware of a nearly 19% recurrence rate that requires reoperation, which occurs most frequently following isolated anterior and lateral compartment releases.^64^ For patients who decline surgery, normal daily functioning is entirely possible, though their participation in strenuous sports will remain restricted.^66^

Ultimately, the prognosis for both conditions is generally excellent when managed appropriately (Table 4).

**Table 4.** Clinical characteristics and outcomes of neonatal compartment syndrome.

Condition or fracture pattern	Prevalence/Incidence	Key diagnostic indicators	Treatment/Management protocols	Reported outcomes/Complications	Source
Non-fracture Acute Compartment Syndrome (Trauma, IV infiltration, infection, iatrogenic).	Up to 30% of all ACS cases; includes ICU patients with obtunded sensorium.	Mean time to diagnosis >48 hours; symptoms include pain (85%), swelling (72%), and tense compartments.	Prompt surgical decompression (fasciotomy) and treatment of underlying cause (e.g., infection control).	Poorer outcomes than fracture-associated ACS; 54% muscle necrosis; 25-31% persistent deficits.	^2,9,20,33,41^
Iatrogenic causes (Traditional Bone Setting).	39.6% to 65.3% of major amputations in specific series.	Intense pain out of proportion; black/gangrenous limb; cold and pulseless extremity.	Urgent fasciotomy and debridement; often requires limb amputation (shoulder or forearm).	High rate of amputation, sepsis, and lifelong disability.	^14,21,46^
Neonatal forearm compartment syndrome.	24 cases over 20 years.	Sentinel bullous or ulcerative skin lesion on dorsal forearm/wrist; distal edema.	Emergency fasciotomy (rarely performed early); late cases require reconstructive surgery.	Volkmann’s contracture, muscle loss, and bone growth abnormalities.	^47^

Despite the profound capacity for recovery in children, the ultimate goal for PACS remains unchanged: early recognition and emergent surgical decompression are essential to saving the limb and ensuring a complete return to normal function.^8,10,15^ Conversely, for patients who decline surgical intervention for CECS, normal daily functioning is entirely possible, though their participation in strenuous sports will remain significantly restricted.^66^

## References

[1] Livingston KS Glotzbecker MP Shore BJ . Pediatric Acute Compartment Syndrome. J Am Acad Orthop Surg 2017; 25(5): 358–364. 10.5435/JAAOS-D-15-00655 28323644

[2] Lin JS Samora JB . Pediatric acute compartment syndrome: a systematic review and meta-analysis. J Pediatr Orthop B 2020; 29(1): 90–96. 10.1097/BPB.0000000000000593 30688754

[3] Gottlieb M Adams S Landas T . Current Approach to the Evaluation and Management of Acute Compartment Syndrome in Pediatric Patients. Pediatr Emerg Care 2019; 35(6): 432–437. 10.1097/PEC.0000000000001855 31157749

[4] Rademacher E Miller PE Jordan E , et al. Management of Fasciotomy Incisions After Acute Compartment Syndrome: Is Delayed Primary Closure More Feasible in Children Compared With Adults? J Pediatr Orthop 2020; 40(4): e300–e305. 10.1097/BPO.0000000000001492 31876698

[5] Yuan PS Pring ME Gaynor TP , et al. Compartment syndrome following intramedullary fixation of pediatric forearm fractures. J Pediatr Orthop 2004; 24(4): 370–375. 10.1097/00004694-200407000-00005 15205617

[6] Pallan TG Elkins JT Dunham AM , et al. Compartment Syndrome of the Pediatric Upper Extremity: Current Concepts. Children 2025; 12(12): 1696. 10.3390/children12121696 41462836 PMC12731944

[7] Hosseinzadeh P Talwalkar VR . Compartment Syndrome in Children: Diagnosis and Management. Am J Orthop 2016; 45(1): 19–22. 26761913

[8] Flynn JM Bashyal RK Yeger-McKeever M , et al. Acute Traumatic Compartment Syndrome of the Leg in Children: Diagnosis and Outcome. J Bone Jt Surg-Am 2011; 93(10): 937–941. 10.2106/JBJS.J.00285 21593369

[9] Livingston K Glotzbecker M Miller PE , et al. Pediatric Nonfracture Acute Compartment Syndrome: A Review of 39 Cases. J Pediatr Orthop 2016; 36(7): 685–690. 10.1097/BPO.0000000000000526 26019026

[10] Lin J Samora WP Samora JB . Acute compartment syndrome in pediatric patients: a case series. J Pediatr Orthop B 2022; 31(2): e236–e240. 10.1097/BPB.0000000000000864 33741835

[11] Ferlic PW Singer G Kraus T , et al. The acute compartment syndrome following fractures of the lower leg in children. Injury 2012; 43(10): 1743–1746. 10.1016/j.injury.2012.06.025 22795846

[12] Sees JA Cutler GJ Ortega HW . Risk Factors for Compartment Syndrome in Pediatric Trauma Patients. Pediatr Emerg Care 2020; 36(3): e115–e119. 10.1097/PEC.0000000000001636 30335686

[13] Cugola L Fasolo G . Clinical and demographic profile Volkmann’s ischemic contracture presenting in Tigray (Ethiopia). Acta Biomed Atenei Parm 2022; 92(S3): e2021562. 10.23750/abm.v92iS3.12557 PMC9437668 35604260

[14] Winanto ID Tirta C . Management of dead limb following traditional practices in Indonesia: A case report. Int J Surg Case Rep 2024; 123(C): 110215. 10.1016/j.ijscr.2024.110215 39232349 PMC11403397

[15] Broom A Schur MD Arkader A , et al. Compartment syndrome in infants and toddlers. J Child Orthop 2016; 10(5): 453–460. 10.1007/s11832-016-0766-0 27538943 PMC5033785

[16] Shore BJ Glotzbecker MP Zurakowski D , et al. Acute Compartment Syndrome in Children and Teenagers With Tibial Shaft Fractures: Incidence and Multivariable Risk Factors. J Orthop Trauma 2013; 27(11): 616–621. 10.1097/BOT.0b013e31828f949c 23481923

[17] Bae DS Kadiyala RK Waters PM . Acute compartment syndrome in children: contemporary diagnosis, treatment, and outcome. J Pediatr Orthop 2001; 21(5): 680–688. 11521042

[18] Souder CD Yang S Greenhill DA , et al. Pediatric Acute Compartment Syndrome. J Pediatr Orthop Soc N Am 2021; 3(2): 252. 10.55275/JPOSNA-2021-252

[19] Machaku D Lodhia J Nangole A , et al. Loss of a limb: A consequence of traditional bone setting. Int J Surg Case Rep 2025; 130(C): 111237. 10.1016/j.ijscr.2025.111237 40168878 PMC11999223

[20] Bhogadi SK El-Qawaqzeh K Colosimo C , et al. Pediatric Acute Compartment Syndrome in Long Bone Fractures: Who is at Risk? J Surg Res 2024; 298: 53–62. 10.1016/j.jss.2024.01.032 38569424

[21] Mashru RP Herman MJ Pizzutillo PD . Tibial Shaft Fractures in Children and Adolescents. J Am Acad Orthop Surg 2005; 13(5): 345–352. 10.5435/00124635-200509000-00008 16148360

[22] Malhotra K Pai S Radcliffe G . Do minimally displaced, closed tibial fractures in children need monitoring for compartment syndrome? Injury 2015; 46(2): 254–258. 10.1016/j.injury.2014.04.046 24972494

[23] Villarreal ED Wrenn JO Sheffer BW , et al. Do Patient-specific or Fracture-specific Factors Predict the Development of Acute Compartment Syndrome After Pediatric Tibial Shaft Fractures? J Pediatr Orthop 2020; 40(3): e193–e197. 10.1097/BPO.0000000000001410 31157755

[24] Percival TJ White JM Ricci MA . Compartment Syndrome in the Setting of Vascular Injury. Perspect Vasc Surg Endovasc Ther 2011; 23(2): 119–124. 10.1177/1531003511401422 21502109

[25] Lyden SP Shortell CK Illig KA . Reperfusion and compartment syndromes: Strategies for prevention and treatment. Semin Vasc Surg 2001; 14(2): 107–113. 10.1053/svas.2001.23166 11400086

[26] Khoshhal KI Alsaygh EF Alsaedi OF , et al. Etiology of trauma-related acute compartment syndrome of the forearm: a systematic review. J Orthop Surg 2022; 17(1): 342. 10.1186/s13018-022-03234-x PMC9258104 35794574

[27] Caba B Susanu SP Caba IC , et al. Paediatric Snakebite Toxicity up to Compartment Syndrome: A Ten-Year Retrospective Study in Iasi, Romania. Toxins 2026; 18(3): 128. 10.3390/toxins18030128 41893551 PMC13030228

[28] Bussell HR Aufdenblatten CA Subotic U , et al. Compartment pressures in children with normal and fractured lower extremities. Eur J Trauma Emerg Surg 2019; 45(3): 493–497. 10.1007/s00068-019-01082-9 30715553

[29] Tharakan SJ Subotic U Kalisch M , et al. Compartment Pressures in Children With Normal and Fractured Forearms: A Preliminary Report. J Pediatr Orthop 2016; 36(4): 410–415. 10.1097/BPO.0000000000000471 25851687

[30] Hahn G . Johns Hopkins All Children’s Hospital Compartment Syndrome Clinical Pathway. Published online. https://share.google/fnjGd3nQoHuMdwuoz (2020).

[31] Kalyani BS Fisher BE Roberts CS , et al. Compartment Syndrome of the Forearm: A Systematic Review. J Hand Surg 2011; 36(3): 535–543. 10.1016/j.jhsa.2010.12.007 21371630

[32] Mubarak SJ . Extensor retinaculum syndrome of the ankle after injury to the distal tibial physis. J Bone Joint Surg Br 2002; 84-B(1): 11–14. 10.1302/0301-620X.84B1.0840011 11837815

[33] Haumont T Gauchard GC Zabee L , et al. Extensor retinaculum syndrome after distal tibial fractures: anatomical basis. Surg Radiol Anat 2007; 29(4): 303–311. 10.1007/s00276-007-0215-3 17502984

[34] Bergen MA Vutescu ES McKinnon S , et al. Risk of Acute Compartment Syndrome in Pediatric Patients With Tibial Tubercle Avulsion Fractures: A Retrospective Review. J Pediatr Orthop 2024; 44(9): 555–560. 10.1097/BPO.0000000000002744 38853742

[35] Milner JD Bergen MA Zhang H , et al. Epidemiology of Acute Compartment Syndrome After Pediatric Tibial Tubercle and Tibial Shaft Fractures. J Pediatr Orthop 2025; 45(3): 134–138. 10.1097/BPO.0000000000002855 39482931

[36] Janzing H Broos P Rommens P . Compartment syndrome as a complication of skin traction in children with femoral fractures. J Trauma 1996; 41(1): 156–158. 10.1097/00005373-199607000-00028 8676413

[37] Noonan KJ McCarthy JJ . Compartment Syndromes in the Pediatric Patient. J Pediatr Orthop 2010; 30(Supplement 2): S96–S101. 10.1097/BPO.0b013e3181d07118

[38] Blakemore LC Cooperman DR Thompson GH , et al. Compartment Syndrome in Ipsilateral Humerus and Forearm Fractures in Children. Clin Orthop 2000; 376: 32–38. 10.1097/00003086-200007000-00006 10906855

[39] Silas SI Herzenberg JE Myerson MS , et al. Compartment syndrome of the foot in children. J Bone Jt Surg 1995; 77(3): 356–361. 10.2106/00004623-199503000-00004 7890783

[40] Wallin K Nguyen H Russell L , et al. Acute Traumatic Compartment Syndrome in Pediatric Foot: A Systematic Review and Case Report. J Foot Ankle Surg 2016; 55(4): 817–820. 10.1053/j.jfas.2016.02.010 27067201

[41] Livingston KS Meehan WP Hresko MT , et al. Acute Exertional Compartment Syndrome in Young Athletes: A Descriptive Case Series and Review of the Literature. Pediatr Emerg Care 2018; 34(2): 76–80. 10.1097/PEC.0000000000000647 27248777

[42] Prasarn ML Ouellette EA Livingstone A , et al. Acute pediatric upper extremity compartment syndrome in the absence of fracture. J Pediatr Orthop 2009; 29(3): 263–268. 10.1097/BPO.0b013e31819c3d54 19305277

[43] Cherry I Franck D Urbain F . Neonatal Limb Compartment Syndrome: A Comprehensive Review. J Hand Surg 2025; 50(3): 379.e1–379.e10. 10.1016/j.jhsa.2023.08.013 38069947

[44] Ball GA Thompson EA Bomar JD , et al. Pediatric Acute Compartment Syndrome in Midshaft Tibia Fractures: Risk Factors for Overnight Observation. J Pediatr Orthop 2026, Published online April 14. 10.1097/BPO.0000000000003291 41979152

[45] Currey JD Butler G . The mechanical properties of bone tissue in children. J Bone Joint Surg Am 1975; 57(6): 810–814. 1158919

[46] Martinez VH Pluta N Tadlock JC , et al. The Prevalence of Acute Compartment Syndrome in Pediatric Tibial Tubercle Fractures. J Pediatr Orthop 2024; 44(10): e883–e886. 10.1097/BPO.0000000000002776 39021084

[47] Hoellwarth JS Geffner A Fragomen AT , et al. Avoiding Compartment Syndrome, Vascular Injury, and Neurologic Deficit in Tibial Osteotomy: An Observational Study of 108 Limbs. JAAOS Glob Res Rev 2023; 7(11). 10.5435/JAAOSGlobal-D-23-00075 PMC10656085 37973030

[48] Ojike NI Alla SR Battista CT , et al. A single volar incision fasciotomy will decompress all three forearm compartments: A cadaver study. Injury 2012; 43(11): 1949–1952. 10.1016/j.injury.2012.08.006 22906919

[49] Jauregui JJ Yarmis SJ Tsai J , et al. Fasciotomy closure techniques. J Orthop Surg (Hong Kong) 2017; 25(1): 2309499016684724. 10.1177/2309499016684724 28176601

[50] Kakagia D . How to close a limb fasciotomy wound: an overview of current techniques. Int J Low Extrem Wounds 2015; 14(3): 268–276. 10.1177/1534734614550310 25256284

[51] Cohn BT Shall J Berkowitz M . Forearm fasciotomy for acute compartment syndrome: a new technique for delayed primary closure. Orthopedics 1986; 9(9): 1243–1246. 3763495 10.3928/0147-7447-19860901-15

[52] Dahners LE . The running near-near-far-far stitch for closure of fasciotomies and other large wounds. Orthopedics 2003; 26(4): 383–384. 12722908 10.3928/0147-7447-20030401-15

[53] Webster J Scuffham P Stankiewicz M , et al. Negative pres sure wound therapy for skin grafts and surgical wounds heal ing by primary intention. Cochrane Database Syst Rev 2014; 10: Cd009261. 10.1002/14651858.CD009261.pub3 25287701

[54] Gwinn MC McGuffin A . Bilateral Exertional Compartment Syndrome With Endoscopic Fasciotomy Surgical Intervention in a High School Athlete. Cureus 2021; 13: e13327, Published online February 13, 2021. 10.7759/cureus.13327 33738170 PMC7958553

[55] Shadgan B Menon M Sanders D , et al. Current thinking about acute compartment syndrome of the lower extremity. Can J Surg 2010; 53(5): 329–334. 20858378 PMC2947124

[56] Argenta LC Morykwas MJ . Vacuum-assisted closure: a new method for wound control and treatment: clinical experience. Ann Plast Surg 1997; 38(6): 563–576, discussion 577. 9188971

[57] Janzing HM Broos PL . Dermatotraction: an effective technique for the closure of fasciotomy wounds: a preliminary report of fifteen patients. J Orthop Trauma 2001; 15(6): 438–441. 10.1097/00005131-200108000-00010 11514772

[58] Caruso DM King TJ Tsujimura RB , et al. Primary closure of fasciotomy incisions with a skin-stretching device in patients with burn and trauma. J Burn Care Rehabili 1997; 18(2): 125–132. 10.1097/00004630-199703000-00006 9095421

[59] Kanj WW Gunderson MA Carrigan RB , et al. Acute compartment syndrome of the upper extremity in children: Diagnosis, management, and outcomes. J Child Orthop 2013; 7(3): 225–233. 10.1007/s11832-013-0492-9 24432081 PMC3672459

[60] Von Keudell AG Weaver MJ Appleton PT , et al. Diagnosis and treatment of acute extremity compartment syndrome. The Lancet 2015; 386(10000): 1299–1310. 10.1016/S0140-6736(15)00277-9 26460664

[61] Buerba RA Fretes NF Devana SK , et al. Chronic exertional compartment syndrome: current management strategies. Open Access J Sports Med 2019; 10: 71–79. 10.2147/OAJSM.S168368 31213933 PMC6537460

[62] Tarabishi MM Almigdad A Almonaie S , et al. Chronic Exertional Compartment Syndrome in Athletes: An Overview of the Current Literature. Cureus 2023; 15: e47797, Published online October 27. 10.7759/cureus.47797 38022185 PMC10676709

[63] Velasco TO Leggit JC . Chronic Exertional Compartment Syndrome: A Clinical Update. Curr Sports Med Rep 2020; 19(9): 347–352. 10.1249/JSR.0000000000000747 32925373

[64] Beck JJ Tepolt FA Miller PE , et al. Surgical Treatment of Chronic Exertional Compartment Syndrome in Pediatric Patients. Am J Sports Med 2016; 44(10): 2644–2650. 10.1177/0363546516651830 27365374

[65] van Heeswijk K Heijmans M van der Cruijsen M , et al. Association Between Number of Positive Pedowitz Criteria and Outcome After a Fasciotomy for Lower Leg Chronic Exertional Compartment Syndrome. Orthop J Sports Med 2026; 14(3): 23259671251394375. 10.1177/23259671251394375 41884701 PMC13009743

[66] García-Mata S . Chronic Exertional Compartment Syndrome of the Forearm in Adolescents. J Pediatr Orthop 2013; 33(8): 832–837. 10.1097/BPO.0b013e3182a0078b 23965913

